# Mucopolysaccharidosis Type II: A Kenyan Case Series

**DOI:** 10.1155/2021/2328402

**Published:** 2021-12-22

**Authors:** L. N. Wainaina Mungai, C. M. Njeru, L. A. Nyamai, M. Maina

**Affiliations:** ^1^University of Nairobi, Nairobi, Kenya; ^2^Kenyatta University, Nairobi, Kenya; ^3^KEMRI Wellcome Trust Research Programme, Kilifi, Kenya

## Abstract

Hunter syndrome, or mucopolysaccharidosis type 2 (MPS2), is a lysosomal storage disorder associated with the involvement of multiple organs such as the central nervous system, hepatomegaly, musculoskeletal, respiratory, cardiac, and hearing. This is due to the accumulation of glycosaminoglycans in body tissues leading to organ failure. Since the laboratories in Kenya do not screen for metabolic diseases, there is the likelihood of assumption that these patients do not exist. These first cases were referred from the eastern part of Kenya where the majority of inhabitants are from the same ethnic community. It was noted that there was increased mortality among boys below the age of 20 years, and hence, the families sought for help in the national referral and teaching hospital. The case series is meant to show that these cases exist and the majority of the patients may be dying before the diagnosis is made. There are no data on MPS2 from Kenya, and the prevalence and incidence are unknown. In this retrospective study, we present a case series of 6 Kenyan boys with MPS2 from a national referral hospital. They were part of 17 patients who had had their blood analyzed for metabolic diseases. All of them were symptomatic with varying degrees of central nervous system involvement. They had undetectable levels of iduronate-2-sulfatase (I2S) enzyme, and three genetic mutations were detected in the IDS gene.

## 1. Introduction

Mucopolysaccharidosis type II (Hunter syndrome or MPS2) is an X-linked disorder caused by a deficiency in the lysosomal enzyme iduronate-2-sulfatase (I2S) leading to the accumulation of glycosaminoglycans (GAGs), dermatan sulfate and heparan sulfate. Accumulation of GAGs within lysosomes leads to progressive cellular damage, subsequent organ failure, and reduced life expectancy [1]. Its incidence is 0.3–0.71 per 100,000 live births [2]. MPS2 presents as either severe or attenuated form depending on the degree of central nervous system involvement. Death of patients with the severe form usually occurs in the first or second decade of life mostly due to cardiopulmonary complications. Mild or attenuated Hunter syndrome is characterized by preservation of intelligence, somatic involvement, and survival into adulthood [3, 4].

The MPS2 patients experience a spectrum of progressive, multiorgan clinical symptoms. The age at presentation and progression of disease can vary. They can have respiratory symptoms in the first months of life but usually present between 2 and 4 years of age. They have an inguinal or umbilical hernia, short stature, coarse facial facies, enlarged tongue, and gum hypertrophy. Other symptoms they can present with are recurrent ear and respiratory infections, obstructive sleep apnoea, dysostosis multiplex, macrocephaly, kyphosis, thickening of the long bone diaphysis, and barrel chest. They also have hepatosplenomegaly, hearing loss, and cardiomyopathy [1, 5].

About two-thirds of patients have psychomotor retardation, behavioral disturbances, and developmental regression. Patients with attenuated form present later in life with minimal neurological dysfunction [1, 2, 6]. They have normal intelligence and can survive into adulthood. The patients with the severe, neuropathic form of Hunter syndrome may have a primary disease with parenchymal neural cognitive impairment due to deposition of GAGs in neural tissue and from other pathophysiologic, neurotoxic, and inflammatory disease mechanisms [7, 8].

Analysis of urine GAGs can be used to confirm the suspicion of Hunter syndrome. Excess urinary excretion of dermatan sulfate and heparan sulfate is a characteristic of Hunter syndrome but not diagnostic as these GAGs can also be elevated in other types of mucopolysaccharidoses. Thus, measurement of I2S enzyme activity is necessary to confirm the diagnosis. Absent or low I2S activity in males is diagnostic of MPS2, but absolute enzyme activity is unable to predict phenotype severity [2, 9, 10]. Genetic testing of IDS allows the prediction of the phenotype and confirms MPS2 in males [5]. Mutations resulting in the complete absence of IDS or its activity are associated with MPS2 with neurologic involvement [2].

Kenya, like many sub-Saharan countries, does not routinely screen for metabolic diseases. Therefore, with a paucity of data on MPS2 in the majority of these countries, most of the patients with severe Hunter syndrome die early before the diagnosis is made.

To our knowledge, no published data exist on MPS2 from Kenya and sub-Saharan Africa. In this paper, we present 6 confirmed cases of MPS2 and describe the clinical characteristics. This preliminary analysis will aid in informing Kenyan healthcare professionals on the presence of MPS2 patients among patients who report having had male relatives who died of similar illness without a diagnosis. It will also form a basis for a larger study among MPS2 patients.

## 2. Methods

This is a descriptive case series of patients receiving care for MPS2 at Kenyatta National Hospital (KNH), the main referral hospital in Kenya. This case report is part of a larger ongoing study seeking to identify the MPS2 carrier rate among relatives of patients with mucopolysaccharidosis type II. Records of patients who had been screened for inborn errors of metabolism and other genetic mutations were retrieved. Data on sociodemographic characteristics, clinical history, and genetic analysis information were extracted and tabulated. A description of the patient characteristics was also conducted.

## 3. Results

Out of the total of 17 children that were reviewed, 6/17 (35.3%) patients had pathogenic variants in the IDS gene associated with X-linked recessive mucopolysaccharidosis type II. They were all symptomatic with mainly neurological, musculoskeletal, and respiratory symptoms. The mean age at diagnosis was 7.5 years with a range of 3–13 years. The guardians noted the first early symptoms at an average age of three years. The six patients were from a total of four families with some being siblings. From the genetic analysis, three different gene mutations were found among the 6 patients. Three of the patients had nonsense mutations, two had missense mutation, and one patient had frameshift mutation.

### 3.1. Case 1

The boy had a missense type of mutation with a severe type of MPS2. He developed the symptoms of nocturnal snoring by age 4 years and then progressively had regression of milestones, and by the age of ten years, he was deaf, could only mumble some things, was hyperactive, and could not follow any instructions. He was snoring when both awake and asleep. He could neither feed nor dress himself. Though he could not be toilet trained, he remained ambulant. He had dysostosis multiplex, hepatomegaly, large umbilical hernia, enlarged tongue, and coarse facial facies. He had macrocephaly, macroglossia, and gingival hypertrophy. There was no history of similar illnesses in the family.

### 3.2. Case 2

He had a severe form of MPS2 with a similar mutation as in case 1. He started snoring while asleep at the age of 3 years. Though he could pronounce some words at the age of 1.5 years, he had regression of speech development, and by 3 years, he could only communicate by pointing at things. He was partially deaf by 5 years of age with dysostosis multiplex. He had the diagnosis confirmed at the age of five years. Though he progressively became aggressive and hyperactive by 7 years of age, he could feed and dress by himself. An older brother with a similar condition had died at 13 years, and from the extended family, 12 boys had died before 15 years of age.

### 3.3. Cases 3, 4, and 5

They were three siblings. They had the same nonsense mutation. Though the enzyme activity for the three patients was the same, the first born had a severe form of the disease with deafness and poor cognitive function. The second sibling was deaf but had good cognitive functions with good school performance. The third sibling was still young and, by the time of diagnosis, had no obvious central nervous system manifestations of the disease. All three had short stature with dysostosis multiplex. They all had course features. Despite being siblings with the same mutation, they had different forms of central nervous system involvement. A maternal uncle had died at 20 years with similar illness.

### 3.4. Case 6

Patient number 6 was an older boy. He had the flame shift type of mutation. By the time the diagnosis was confirmed, he was both mentally and physically incapacitated. He could neither feed himself nor walk. He had signs and symptoms of severe disease. He presented with respiratory symptoms characterized by dyspnoea at rest and sleep obstructive apnoea. An older brother and two maternal cousins had died of similar illnesses at the age of 13 and 14 years.

An older brother with a similar condition had died at 13 years, and from the extended family, 12 boys had died before 15 years of age (Table 1).

## 4. Discussion

The three siblings with a nonsense mutation had IDS variant. This was previously described as a mutation that causes mucopolysaccharidosis II [9, 11, 12]. Cases 1 and 2 had missense mutation, and case 6 had frameshift mutation. This is similar to other MPS2 studies. In 2016, Kosuga et al. analyzed mutations in the IDS gene of 65 patients suffering from MPS II in the Japanese population. They were diagnosed with both the accumulation of urinary glycosaminoglycans and a decrease in their IDS enzyme activity. Among the samples examined, they identified different mutations: 33 missense, 8 nonsense, 7 frameshift, and 4 intronic changes. Consistent with the previous studies, the Japanese study showed that most of the attenuated phenotypes were derived from the missense mutations of the IDS gene, whereas mutations associated with a large alteration which included splicing, frameshift, and nonsense mutations were linked to the severe phenotype of MPS II [9].

The three siblings had varying clinical presentations. This has been found in other studies where they looked at sibling pairs. Ficicioglu et al. looked at the data in the Hunter Outcome Survey to study the intrafamilial variability in the clinical manifestation of MPS II in 2017. They found that a majority of sibling pairs had a similar phenotypic presentation. However, there was discordance in some areas; out of 28 sibling pairs, four had different intelligent quotient that was used to measure the cognitive function. The mental function was rated as normal or borderline, educable, trainable, or profound. They also found differences in regression in walking in 8 pairs, speech in 8 pairs, dress alone in one pair, and toilet training regression in 1 pair. This has been suggested for the differences between siblings. After looking at the clinical presentations at different ages, Ficicioglu et al. concluded that the chronological age difference between siblings may not have been the cause of discordance [13]. In our study, the sibling who was least affected was the youngest at 3 years. The oldest sibling was the most affected. He was not able to learn which may have been affected by the loss of hearing. However, the middle sibling was also deaf but was able to overcome it by using his eyes to follow the teacher. Clinical variability among siblings with the same condition has been found in other lysosomal storage diseases (LSDs) such as Pompe disease, Fabry disease, and Sanfilippo syndrome. There have been suggestions to indicate that epigenetic and genetic may be the cause [14–17].

The two patients with a missense mutation, that is, cases 1 and 2, presented with rapidly progressing severe type of MPS2. This is contrary to the Japanese study where the patients with similar mutations had the attenuated form of MPS2. Case 6 presented at 13 years of age when he was already moribund. He had a neuropathic form of the disease with severe somatic symptoms. He snored while awake and asleep and was not aware of his surroundings. The Japanese study associated frameshift mutation with severe disease [13].

## 5. Conclusion

Our patients had varying clinical presentations, similar to patients in previous studies. The 6 cases in our study are too few to conclude on the clinical presentations and types of mutations; however, they give an idea of the local phenotype and IDS gene mutations in Kenyan MPS2 patients.

## Figures and Tables

**Table 1:** Clinical characteristics of mucopolysaccharidosis type II patients at Kenyatta National Hospital.

Patient	Case 1	Case 2	Case 3	Case 4	Case 5	Case 6
Age in years when the guardian noted symptoms	4	3	3	3	3	3
Age in years at diagnosis	10	5	15	13	3	3
CNS symptoms and signs	Hyperactivity, poor cognitive function, irritability, not talking	Hyperactivity and irritability, poor cognitive function, mumbles things	Attention-deficit, poor cognitive function, language intact	Very intelligent despite being deaf, good school performance	Functionally normal	Severe CNS dysfunction
Respiratory symptoms	Enlarged tongue, gum hypertrophy, dyspnoea, sleep apnoea	Enlarged tongue, gum hypertrophy, dyspnoea, sleep apnoea	Enlarged tongue, gum hypertrophy, dyspnoea, sleep apnoea	Enlarged tongue, gum hypertrophy, dyspnoea, sleep apnoea	Snoring when asleep	Enlarged tongue, gum hypertrophy, dyspnoea, sleep apnoea
Abdomen	Umbilical hernia, hepatomegaly	Umbilical hernia, hepatomegaly	Umbilical hernia, hepatomegaly	Umbilical hernia, hepatomegaly	Umbilical hernia, hepatomegaly	Umbilical hernia, hepatomegaly
Ears	Recurrent otitis media and deaf	Recurrent otitis media and deaf	Recurrent otitis media and deaf	Recurrent otitis media and deaf	None	Recurrent otitis media and deaf
Skeletal	Carpal tunnel syndrome, multiple joint contractures	Carpal tunnel syndrome, multiple joint contractures	Carpal tunnel syndrome, multiple joint contractures	Carpal tunnel syndrome, multiple joint contractures	Carpal tunnel syndrome	Carpal tunnel syndrome, multiple joint contractures
Iduronate-2-sulfatase level, ref ≥5.6 *μ*mol/L/h	<0.8 *μ*mol/L/h	<0.8 *μ*mol/L/h	<0.8 *μ*mol/L/h	<0.8 *μ*mol/L/h	<0.8 *μ*mol/L/h	<0.8 *μ*mol/L/h
Gene mutation type	Missense	Missense	Nonsense	Nonsense	Nonsense	Frameshift

## Data Availability

The laboratory reports and photos of patients data used to support the findings of this study have not been made available because of patient privacy.

## References

[1] Martin R., Beck M., Eng C. (2008). Recognition and diagnosis of mucopolysaccharidosis II (hunter syndrome). *Pediatrics*.

[2] Neufeld E. F. (2001). The mucopolysaccharidoses. *The Metabolic and Molecular Bases of Inherited Disease*.

[3] Chen Y. (2001). *The Metabolic and Molecular Bases of Inherited Disease*.

[4] Vafiadaki E., Cooper A., Heptinstall L. E., Hatton C. E., Thornley M., Wraith J. E. (1998). Mutation analysis in 57 unrelated patients with MPS II (hunter’s disease). *Archives of Disease in Childhood*.

[5] Wraith J. E., Scarpa M., Beck M. (2008). Mucopolysaccharidosis type II (hunter syndrome): a clinical review and recommendations for treatment in the era of enzyme replacement therapy. *European Journal of Pediatrics*.

[6] Beck M. (2011). Mucopolysaccharidosis type II (hunter syndrome): clinical picture and treatment. *Current Pharmaceutical Biotechnology*.

[7] Holt J. B., Poe M. D., Escolar M. L. (2011). Natural progression of neurological disease in mucopolysaccharidosis type II. *Pediatrics*.

[8] Holt J., Poe M. D., Escolar M. L. (2011). Early clinical markers of central nervous system involvement in mucopolysaccharidosis type II. *The Journal of Pediatrics*.

[9] Kosuga M., Mashima R., Hirakiyama A. (2016). Molecular diagnosis of 65 families with mucopolysaccharidosis type II (hunter syndrome) characterized by 16 novel mutations in the IDS gene: genetic, pathological, and structural studies on iduronate-2-sulfatase. *Molecular Genetics and Metabolism*.

[10] Scarpa M., Almássy Z., Beck M. (2011). Mucopolysaccharidosis type II: European recommendations for the diagnosis and multidisciplinary management of a rare disease. *Orphanet Journal of Rare Diseases*.

[11] Zhang H., Li J., Zhang X. (2011). Analysis of the IDS gene in 38 patients with hunter syndrome: the c.879G>A (p.Gln293Gln) synonymous variation in a female create exonic splicing. *PLoS One*.

[12] Bunge S., Steglich C., Beck M. (1992). Mutation analysis of the iduronate-2-sulfatase gene in patients with mucopolysaccharidosis type II (hunter syndrome). *Human Molecular Genetics*.

[13] Ficicioglu C., Giugliani R., Harmatz P., Mendelsohn N. J., Jego V., Parini R. (2018). Intrafamilial variability in the clinical manifestations of mucopolysaccharidosis type II: data from the hunter outcome survey (HOS). *American Journal of Medical Genetics, Part A*.

[14] Rigoldi M., Concolino D., Morrone A. (2014). Intrafamilial phenotypic variability in four families with Anderson-Fabry disease. *Clinical Genetics*.

[15] Van de Kamp J. J., Niermeijier M. F., Figura K. V., Giesberts M. A. (1981). Genetic heterogeneity and clinical variability in the Sanfilippo syndrome (types A, B, and C). *Clinical Genetics*.

[16] Verovnik F., Benko D., Vujkovac B., Linthorst G. E. (2004). Remarkable variability in renal disease in a large Slovenian family with Fabry disease. *European Journal of Human Genetics*.

[17] Young I. D., Harper P. S., Archer I. M., Newcombe R. G. (1982). A clinical and genetic study of hunter’s syndrome. 1 heterogeneity. *Journal of Medical Genetics*.

